# Safety assessment of multiple repeated percutaneous punctures for the collection of cerebrospinal fluid in rats

**DOI:** 10.1590/1414-431X202010032

**Published:** 2021-04-26

**Authors:** Dongxue Wang, Ying Zhao, Yang Yang, Hailong Xie

**Affiliations:** 1 College of Pharmacy, Heilongjiang University of Chinese Medicine, Harbin, Heilongjiang, China; 2 College of Pharmacy, Harbin University of Commerce, Harbin, Heilongjiang, China

**Keywords:** Cerebrospinal fluid, Safety, Cisterna magna, Brain, Memory and learning test

## Abstract

The objective of this study was to examine the safety of multiple repeated percutaneous punctures of cisterna magna for collecting cerebrospinal fluid (CSF) and preliminarily determine the optimal time interval and volume at each collection. Sixty Wistar rats were randomly assigned to six groups: 10 d-0 μL, 10 d-100 μL (100 μL CSF collected at an interval of 10 days), 10 d-150 μL, 15 d-0 μL, 15 d-100 μL, and 15 d-150 μL. CSF was collected by percutaneous puncture of the cisterna magna at four time-points. Simultaneously, locomotor activity, cisterna magna pressure, and acetylcholine levels in the CSF were monitored. Compared with the 10 d-0 μL group, the escape latency by Morris water maze was significantly prolonged in the 10 d-100 μL and 10 d-150 μL groups (P<0.05). Compared with the 15 d-0 μL group, the indices of 15 d-100 μL and 15 d-150 μL groups had no significant differences. When compared with that at the first training, the exception of the 10 d-150 μL and 15 d-150 μL groups, significant differences in escape latency were found at the 6th attempt (P<0.05). Compared with baseline readings for each group, the cisterna magna pressure in the 10 d-150 μL group began to decrease significantly from the third measurement (P<0.05). The optimal time interval during four CSF collections (100 μL per collection) via cisterna magna percutaneous puncture was determined to be 15 days. The procedure did not significantly affect learning processes, performance, or other related indices.

## Introduction

In animal experiments studying the central nervous system (CNS), cerebrospinal fluid (CSF) samples are often needed to monitor chemical analytes including broad biological markers or neuro-transmitters (1-3). For example, acetylcholine is an important neurotransmitter in the central nervous system. It plays a special role in cognitive functions such as learning and memory. The main pathway of learning and memory is composed of central cholinergic transmission (4–7). Especially in the context of precision medicine, it is necessary to repeatedly collect CSF to successfully describe the dynamic development of a particular disease (8–10).

At present, most researchers adopt microdialysis and transdural puncture of the cisterna magna under direct vision for multiple repeated collections of CSF. First, microdialysis for multiple repeated collections of CSF is safer, but the recovery is low, which is not conducive to the detection of metabolites of some micro-components. At the same time, in order to meet the requirements of the volume for assaying, microdialysis is a time-consuming procedure for CSF collection from a single rat, which has high requirements for the instrument platform and surgery; moreover, it has a high operating cost (11–14).

Second, for transdural puncture of the cisterna magna under direct vision, a longitudinal incision is made along the posterior midline and the dorsal muscles of the neck undergo blunt dissection. The muscles are deeply attached to the bone, which are then scraped with the back of a scalpel. After exposing the atlanto-occipital fascia, a micro-syringe equipped with a tip is used to collect CSF when observing the foramen magnum (15–21). This method is prone to causing an incision infection and is unfavorable for the detection of various indices in the test.

However, most current studies on safety assessment tend to investigate the safety of the collection method, which is based on the mortality and success rate following a one-time CSF collection procedure (15–19). In addition, the assessment indices for the collection volume and safety are unsatisfactory.

Multiple repeated percutaneous punctures of the cisterna magna for CSF collection avoid the shortcomings of the above two methods, with high success rates, minimal trauma, very low incidences of infection, and a very low rate of mortality in treated rats; however, the effects of the time interval and the volume of CSF harvested at each collection for this method on the CNS have up to now not been reported in the literature.

In this setting, this study systematically investigated locomotor activity learning and memory abilities, pressure found in the cisterna magna, and acetylcholine (Ach) levels in the CSF in a rat model system. This study additionally explored cerebral blood flow (CBF) velocity and was developed to preliminarily determine the safety of multiple repeated percutaneous punctures with the aim of collecting CSF of the cisterna magna, optimal time interval, and volume at each collection.

## Material and Methods

### Experimental materials

Pentobarbital sodium was purchased from Sigma (batch number: P11011; USA); scalp needles were provided by Jiangxi Hongda Medical Devices Group Co., Ltd. (batch number: d20160922; China). The rat stereotaxic apparatus was a product of BIOPAC (USA); the Morris water maze was purchased from Huaibei Zhenghua Biologic Apparatus Facilities Co., Ltd. (China); the open field test cage was provided by Huaibei Zhenghua Biological Instrument Equipment Co., Ltd.; the ACQUITY UPLC-I-CLASS was purchased from the Waters Company (USA); the XevoTQD-triple quadrupole mass spectrometer was a product from Waters Company; the MS105 type 1/100,000 analytical balance was purchased from the METTLER TOLEDO Company (Germany); the BL-420 biological function experimental system was provided by Chengdu Taimeng Technology Co., Ltd. (China); and the moorVMS dual-channel laser Doppler flowmeter was purchased from Moor Instruments (UK).

### Experimental animals and grouping

Sixty Wistar rats with an age of 10-12 weeks and a weight of 240±20 g were selected and were divided equally into groups. Animals were provided by Changchun Yisi Experimental Animal Technology Co., Ltd. (China). The protocol approval number was SCXK (Ji)-2016-0003, and the certificate number was 201700017161. Rats were maintained in an individually ventilated cage system (4 rats/cage) with carefully regulated temperature of 22±2°C and controlled humidity of 50±5%.

Animals were randomly assigned into six groups according to body weight and sex. The groups were defined as follows: 10 d-0 μL, 10 d-100 μL (100 μL CSF collected at an interval of 10 days), 10 d-150 μL, 15 d-0 μL, 15 d-100 μL, and 15 d-150 μL.

### CSF collection, measurement of cisterna magna, and CBF

Dynamic measurement of cisterna magna pressure was done in each group. For the 10 d-0 μL, 10 d-100 μL, and the 10 d-150 μL groups, CSF was collected at 0, 10, 20, and 30 d, after which the cisterna magna pressure was measured. For the 15 d-0 μL, 15 d-100 μL, and 15 d-150 μL groups, CSF was collected at 0, 15, 30, and 45 d, after which the cisterna magna pressure was measured.

**Figure 1:**
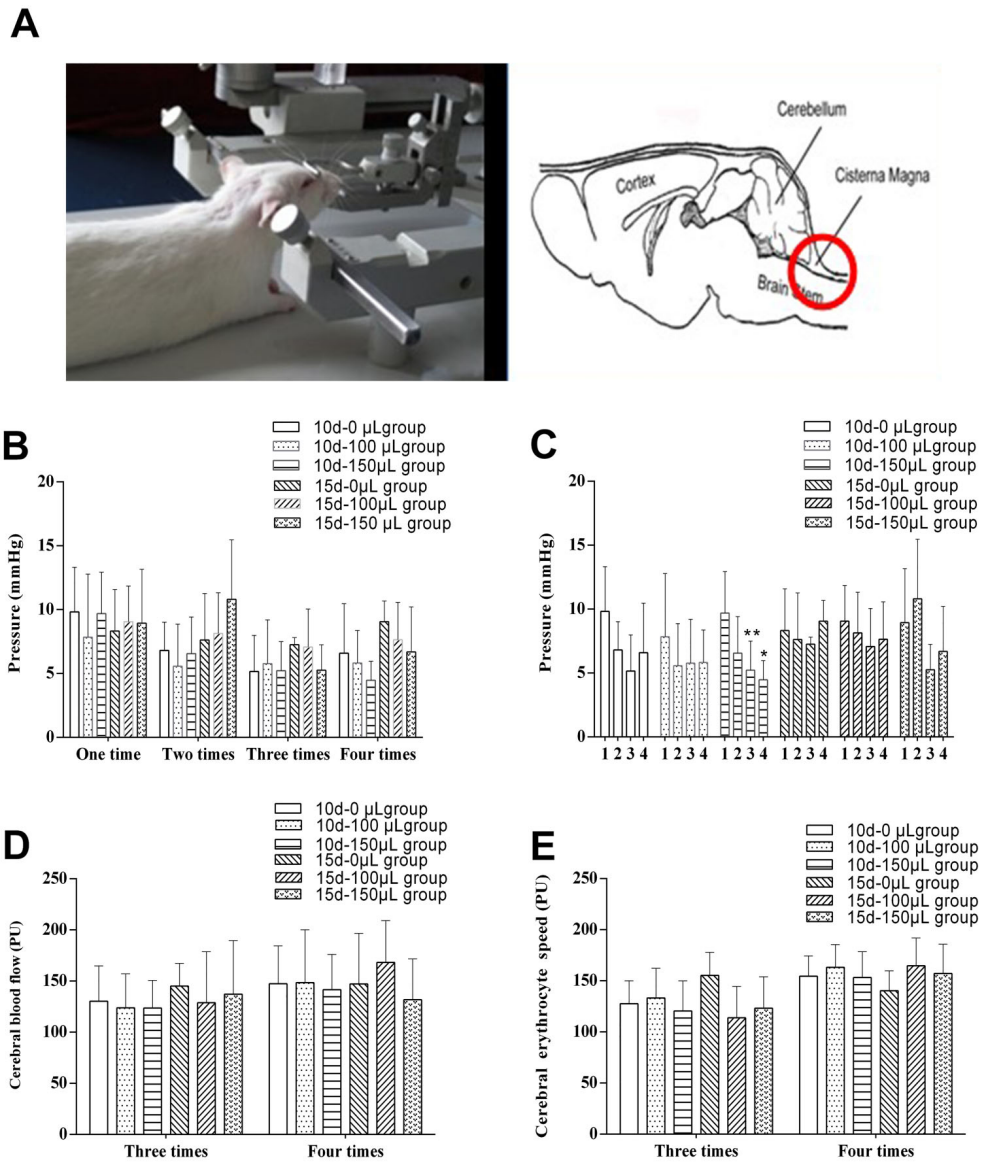
A, Cerebrospinal fluid (CSF) collection device. **B** and **C**, Effect of CSF acquisition on the pressure of cisterna magna in rats; comparison between (**B**) all groups at the time of collection and (**C**) each group over 4 collection time points. **D** and **E**, Effect of CSF acquisition on cerebral blood flow and erythrocyte speed in rat frontal cortex. 10 d-100 μL (interval and volume of CSF collection) group (n=8), 10 d-150 μL group (n=8), 15 d-100 μL group (n=8), and 15 d-150 μL group (n=8) were compared to the control group (n=9). Data are reported as means±SD. *P<0.05, **P<0.01 *vs* the first CSF collection (ANOVA). PU: signal strength unit.

Rats were anesthetized with 3% pentobarbital sodium (40 mg/kg) and fixed on the brain stereotaxic apparatus. The needle was inserted at approximately 0.5 cm at the depression area of the occipital crest to reach the cisterna magna (Figure 1A). Then, the needle insertion was immediately ceased and the other side of the collection needle was connected to the pressure transducer. The BL-420 biological function experiment system was used to monitor the cisterna magna pressure in each group. After 30 s, the CSF was slowly extracted, transferred to a 1.5-mL EP tube, and stored at -80°C. For the 10 d-0 μL and the 15 d-0 μL groups, only the cisterna magna pressure was monitored without CSF collection.

To enable dynamic measurement of CBF in each group, the procedure comprised the following steps. After collecting CSFs measuring the cisterna magna pressure for the third and fourth time-point, data were collected and processed with the moorVMS laser Doppler flowmeter (LDF), and the Doppler fiber-optic probe was then fixed to the frontal lobe for continuous monitoring over a period of 10 min. The average CBF (flux) and erythrocyte speed (speed) were determined every 10 min, and analyzed by the VistaTM software from Moor Instruments (UK).

### Locomotor activity test

For the 10-day groups, the locomotor activity test was performed at 0, 11, 21, and 31 days. For the 15-day groups, the locomotor activity test was performed at 0, 16, 31, and 46 days. The test was performed in an open field cage with dimensions of 100×100×40 cm. Before the test, animals were left for free activities in the open field cage for 5 min, twice per day for two consecutive days, in order to reduce any anxiety that might be experienced by the animals when acclimating to a new environment. During the test, animals were randomly placed into the cage, and the total distance and average speed of the rats in 5 min were recorded using the Xeye SI software program from Huaibei Zhenghua Biological Instrument Equipment Co., Ltd. (China).

### MWM test

The Morris water maze (MWM) test was performed at 32 d in the 10-day groups and at 47 d for the 15-day groups.

The MWM is a circular pool with a diameter of 150 cm and a height of 50 cm. The inner wall of the pool is painted black. The pool was divided into 4 quadrants by 4 equidistant points. Triangle, square, circle, and pentagram card markers were affixed to the center of the 4 quadrant pool walls. A platform was placed in the center of one of the quadrants 2 cm below the water surface, and the water temperature was controlled at 23.0±2°C. At 1 d before the test, the rats were placed in the water maze without the platform to get the animals familiar with the water environment for a period of 90 s. During the test, the rats were randomly placed into the water facing the pool wall in any of the three quadrants that were not the target (i.e., the quadrant where the platform was located), and the time required by the rats to locate the underwater platform after being placed into the water (i.e., the escape latency) was recorded. After the platform was found, animals were allowed to remain there for 10 s. If the rats failed to find the platform within 90 s, the latency was recorded as 90 s, and the rats were guided to stay on the platform for 10 s and then allowed to perform spatial learning and memory test according to the references of the four quadrants. The rats were trained twice daily (4-h interval for each time). Continuous place navigation was conducted for a period of five days and the last training was used as the test period. At day 6, the underwater platform was removed to permit performing the spatial probe test, and the residence time (staying in one particular place) at the target quadrant and the number of times that the animal crossed the platform within 90 s were recorded.

### UPLC-MS/MS for the detection of Ach content in rat CSF

#### Sample pretreatment

The 100-μL sample of CSF was used and supplemented with 400 μL of methanol, then vortexed for 3 min, subjected to ultrasonic treatment for 1 min, and centrifuged at 4360 *g* (4°C) for 10 min. Next, the supernatant was taken and dried at 37°C with nitrogen gas, and the residue dissolved in 60 μL of methanol (equivalent to 1.67-times the concentration of CSF sample). After vortexing for 3 min, the sample was subjected to ultrasonic treatment for 1 min, with centrifugation at 4360 *g* (4°C) for 15 min. Subsequently, the supernatant was taken and a 10-μL sample was analyzed using the ultra-performance liquid chromatography-tandem mass spectrometer (UPLC-MS/MS).

#### LC-MS/MS conditions

The LC-MS/MS analysis was run on an Acquity UPLCTM system. The analytes were separated on an Acquity UPLC TM HSST3 column (2.1×100 mm, 1.7 m, Waters) that was run at a constant temperature of 30°C. The mobile phase, consisting of 0.1% formic acid in water (Solvent A) and acetonitrile (Solvent B), was used under conditions of gradient elution: 0-0.5 min, 10% B; 0.5-1.5 min, 60% B; 1.5-1.75 min, 10% B; 1.75-2 min, and 10% B; at a flow rate of 0.5 mL/min. ESI-MS/MS conditions were set as follows: the instrument was operated using an electrospray ionization source (ESI) in positive mode. ESI parameters were a capillary voltage of 3.0 kV, an extractor voltage of 30 V, a source temperature of 120°C, a desolvation temperature of 350°C, a cone gas flow of 80 L/h, and a desolvation flow of 650 L/h (both gases were nitrogen). Multiple reaction monitoring (MRM) transitions, cone voltages of 28 V, and collision energies of 25 V were applied during the analysis. Data acquisition was performed using Mass Lynx 4.0 software with the Quan Lynx program (obtained from Waters).

#### Methodological investigation

1) Investigation of specificity: according to the chromatographic conditions above, the reference Ach solution was accurately absorbed with an injection of 10 μL of sample and the chromatogram was recorded. The resolution of Ach was observed; 2) Investigation of linear relationship: 1 mg of Ach was dissolved to a constant volume of 5 mL with distilled water to obtain the Ach standard stock solution. Appropriate amounts of standard stock solution were diluted to standard dilutions of 0.3, 0.63, 1.25, 2.5, 5, and 10 ng/mL. The linear regression equation for the peak height Y and the concentration X were calculated. Standard solutions of low, medium, and high concentrations (0.63, 2.5, and 4 ng/mL) were prepared in the same way for the quality control (QC) samples; 3) Investigation of precision: the 0.31, 2.5, and 5.0 ng/mL Ach standard dilutions were used. Each concentration of standard solution was repeatedly measured three times within 1 d; the within-day precision was calculated according to the corresponding peak area of the sample concentration. The same method was used for continuous measurement over three days to calculate the inter-day precision; 4) Investigation of recovery: the Ach standard dilutions of 0.31, 2.5, and 5.0 ng/mL were used for three consecutive injections, and the relative recovery was calculated by taking the ratio of the measured concentration (i.e., the concentration of each sample obtained from the standard curve) to the concentration of the Ach standard solutions; and 5) Investigation of stability: the test solution was used, and 10 μL of the sample was injected at 24 and 48 h, and the relative standard deviation (RSD) of the integral value of the peak area in each component was calculated.

### Statistical analyses

Data analyses were performed with analysis of variance (ANOVA) using the SPSS version 21.0 software program (IBM, USA). The data are reported as means±SD. When data had a normal distribution, the independent Student's *t*-test was used to compare two groups and one-way ANOVA was used for comparisons of multiple groups. When data did not conform to a normal distribution, the comparison among groups was done with the rank sum test. P<0.05 was considered to be a statistically significant effect and a P<0.01 was considered a highly significant effect.

## Results

### Effect of CSF collection on cisterna magna pressure in rats

Compared with the 10d-0 μL group, there was no significant difference in cisterna magna pressure in the 10 d-100 μL or 10 d-150 μL groups (P>0.05). Compared with the 15 d-0 μL group, there was no significant difference in the cisterna magna pressure in the 15 d-100 μL or 15 d-150 μL groups (P>0.05; Figure 1B). Compared with baseline, the cisterna magna pressure in the 10 d-150 μL group began to decrease significantly by the third measurement (P<0.05) and was significantly decreased even more by the fourth measurement (P<0.01). There was no significant difference in cisterna magna pressure among the remaining groups (P>0.05; Figure 1C).

### Effect of CSF collection on CBF in the rat cerebral frontal cortex

Compared with the 10 d-0 μL group, there were no significant differences in CBF velocity and erythrocyte speed in either the 10 d-100 μL group or the 10 d-150 μL group (P>0.05; Figure 1D and E). Compared with the 15 d-0 μL group, there were no significant differences in CBF velocity and erythrocyte speed in either the 15 d-100 μL group or the 15 d-150 μL group (P>0.05; Figure 1D and E).

### Effect of CSF collection on locomotor activity of rats

**Figure 2:**
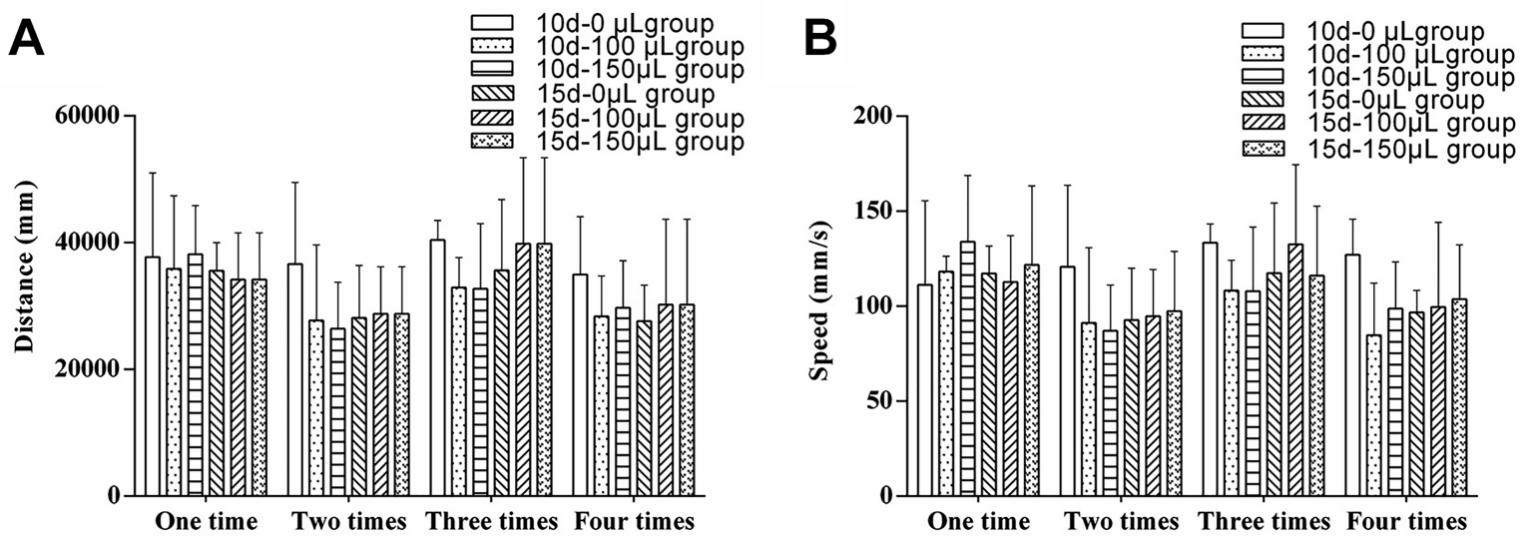
Aand **B**, Effect of cerebrospinal fluid acquisition on the distance and speed of spontaneous activity in rats. 10 d-100 μL (interval and volume of CSF collection) group (n=8), 10 d-150 μL group (n=8), 15 d-100 μL group (n=8), and 15 d-150 μL group (n=8) were compared to the control group (n=9). Data are reported as means±SD (P>0.05, ANOVA).

Compared with the 10 d-0 μL group, there were no significant differences seen in the total distance of locomotor activity and the average moving speed in the 10 d-100 μL and 10 d-150 μL groups (P>0.05; Figure 2A). Compared with the 15 d-0 μL group, no significant differences were seen in the total distance of locomotor activity and average moving speed in the 15 d-100 μL and 15 d-150 μL groups (P>0.05; Figure 2A). Furthermore, compared to the first CSF collection, no significant changes were seen for total distance of locomotor activity and average moving speed over a 5-min time period for any of the groups (P>0.05; Figure 2B).

### Effect of CSF collection on learning and memory abilities of rats

**Figure 3:**
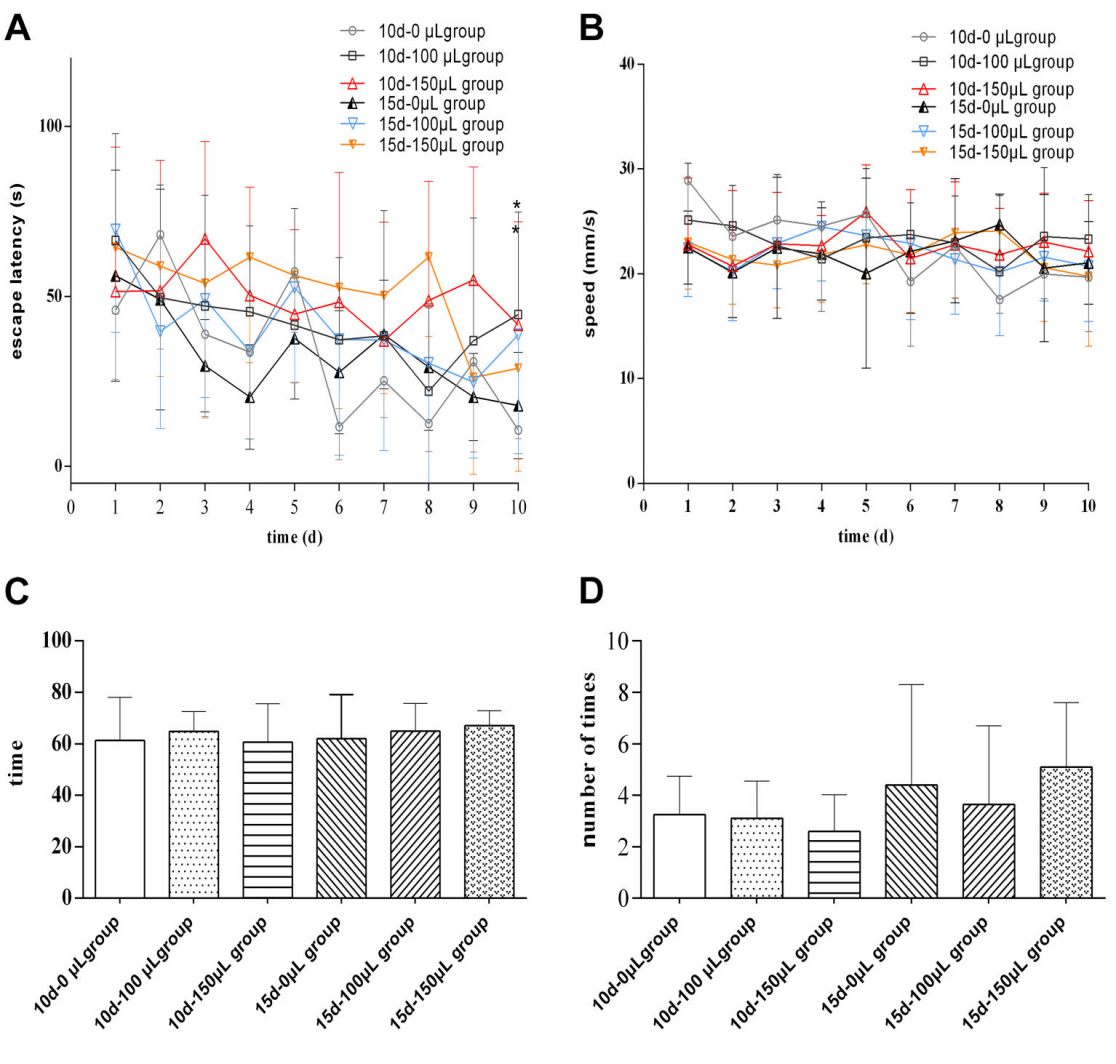
Aand **B**, Effect of cerebrospinal fluid acquisition on learning and memory ability in rats. **C** and **D,** Time spent in target quadrant and number of times rats in each group crossed the platform. 10 d-100 μL (interval and volume of CSF collection) group (n=8), 10 d-150 μL group (n=8), 15 d-100 μL group (n=8), and 15 d-150 μL group (n=8) were compared to the control group (Control, n=9). Data are reported as means±SD. *P<0.05 *vs* 10 d-0 μL group (ANOVA).

Compared with the 10 d-0 μL group, the escape latency during the testing period was significantly prolonged in both the 10 d-100 μL and 10 d-150 μL groups (P<0.05), but there were no significant differences in swimming speed, residence time at the target quadrant, and number of platform crossings (P>0.05; Figure 3A-D). There were no significant differences in the escape latency during the testing period, swimming speed, residence time at the target quadrant, and number of platform crossings between the 15 d-0 μL group and the 15 d-100 μL and 15 d-150 μL groups (P>0.05; Figure 3A-D). When compared to the first training, rats in the 10 d-0 μL, 15 d-0 μL, 10 d-100 μL, and 15 d-100 μL groups showed significant differences in escape latency at the 6th attempt (P<0.05), and for the 15 d-150 μL group at the 9th attempt (P<0.05). However, there was no significant difference for the escape latency during the training period in the 10 d-150 μL group (P>0.05; Table 1).

**Table 1:** Learning and memory ability score in 10 sessions of the test of rats with different intervals and volumes of cerebrospinal fluid collection.

Group	1	2	3	4	5	6	7	8	9	10
10 d-0 μL	63.54±30.72	66.21±34.23	38.51±25	26.74±21.74	51.77±36.98	13.19±10.13ˆˆ	25.82±16.58ˆ	18.54±9.51ˆ	28.14±26.16	13.55±7.4ˆ
10 d-100 μL	66.43±29.85	49.65±31.48	47.19±30.92	45.46±24.01	41.51±32.64	37.24±22.93ˆ	38.42±34.99	22.14±23.33ˆˆ	36.95±34.26ˆ	44.65±28.65*
10 d-150 μL	51.50±40.30	51.68±36.40	66.99±27.12	50.32±30.24	44.77±24.90	48.35±36.15	36.94±33.18	48.79±33.30	54.91±31.57	41.60±28.80*
15 d-0 μL	54.27±25.35	63.16±21.05	32.65±19.71	27.92±23.16	34.79±38.91	21.34±23.36ˆ	32.23±28.98ˆ	26.09±15.24ˆˆ	30.68±24.51ˆ	14.86±12.66ˆˆ
15 d-100 μL	69.56±30.19	39.71±28.58	49.19±28.90	33.75±25.70	52.61±27.88	37.3±34.06ˆ	37.18±32.52ˆ	30.37±37.19ˆ	24.8±22.36ˆˆ	38.71±35.01ˆ
15 d-150 μL	65.05±20.60	55.79±32.42	50.25±39.83	58.73±31.23	52.79±31.17	48.88±35.18	46.27±27.06	60.01±23.99	19.8±20.15ˆˆ	22.09±22.68ˆˆ

Data are reported as mean±standard deviation for all model groups (n=8) and the control group (n=9). *P<0.05 *vs* 10 d-0 μL group. ˆP<0.05, ˆˆP<0.01 *vs* the first training (ANOVA).

### UPLC-MS/MS for detection of Ach content in rat CSF

**Figure 4:**
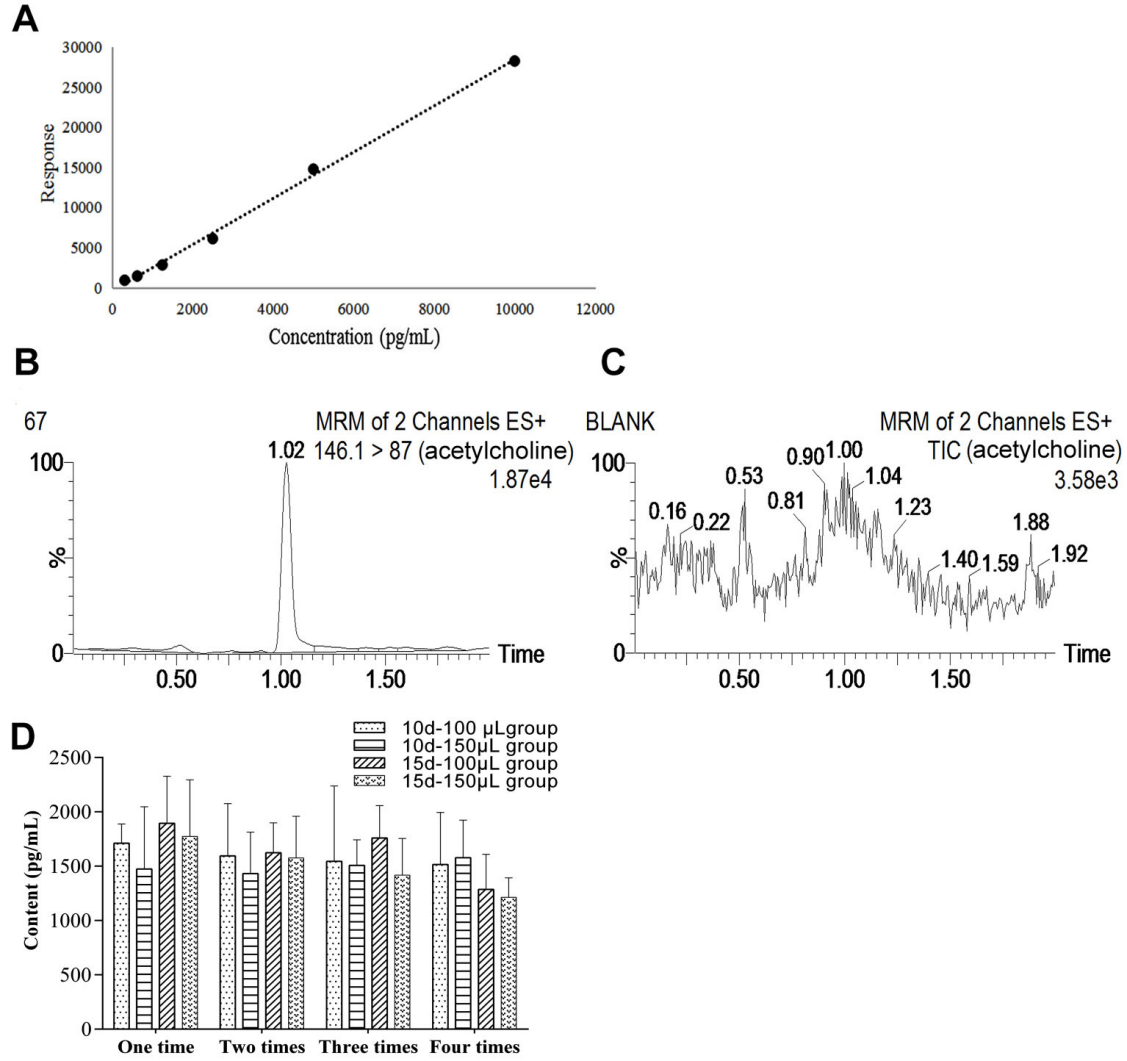
A, Standard curve of acetylcholine. **B** and **C**, Multiple reaction monitoring (MRM) chromatograms of acetylcholine in rat cerebrospinal fluid. **D**, Changes of acetylcholine content in rat cerebrospinal fluid. 10 d-100 μL (interval and volume of CSF collection) group (n=8), 10 d-150 μL group (n=8), 15 d-100 μL group (n=8), and 15 d-150 μL group (n=8) were compared to the control group (n=9). Data are reported as means±SD (P>0.05, ANOVA). Panel B: 67 indicates the serial number of the sample tested. TIC: total ion chromatogram.

The regression equation of Ach was: Y=2.882X-404.04, with an r=0.9996, and a linear range of 0.31-10 ng/mL (Figure 4A). In addition, Ach exhibited better resolution and specificity (Figure 4B and C), and its precision, stability, and recovery were all qualified (RSD <5%; Table 2 and Table 3). Compared to the first collection of CSF, the Ach levels in the rat CSF for each group were similar (P>0.05; Figure 4D).

**Table 2:** Precision and recovery for the determination of the acetylcholine in rat cerebrospinal fluid.

Compound	Concentration (ng/mL)	Intra-day	Inter-day	Recovery
		Precision (RSD, %)	Precision (RSD, %)	
Acetylcholine	0.31	3.0	2.46	1.07
	2.5	2.14	1.59	98.71
	5.0	2.54	2.21	1.08

RSD: relative standard deviation.

**Table 3:** Stability of the acetylcholine in rat cerebrospinal fluid.

Compound	Concentration (ng/mL)	Freeze-thaw cycles		At -80°C for a month		Autosampler for 24 h	
		Accuracy (%)	Precision (RSD, %)	Accuracy (%)	Precision (RSD, %)	Accuracy (%)	Precision (RSD, %)
Acetylcholine	0.31	96.3	3.9	97.8	4.5	99.2	4.2
	2.5	101.7	3.4	98.2	3.9	96.6	3.7
	5.0	97.3	2.8	104.3	3.4	98.5	2.4

RSD: relative standard deviation.

### Optimal acquisition interval and collection volume

**Figure 5:**
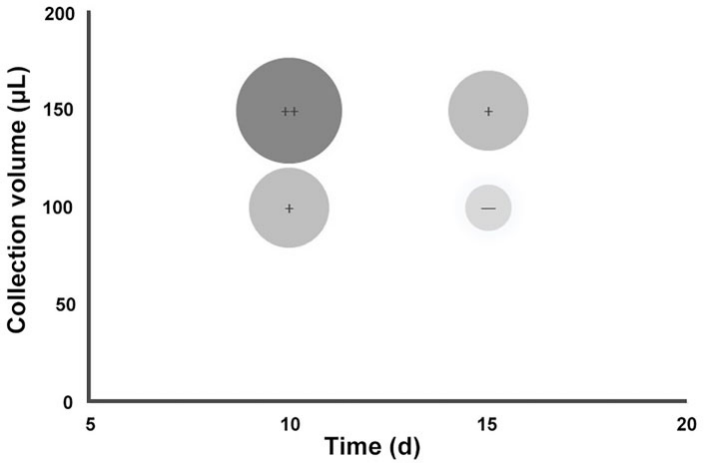
Confirmation of the optimal interval acquisition time and collection volume. Significant changes in locomotor activity, learning and memory abilities, cisterna magna pressure, and Ach levels in the cerebrospinal fluid are denoted by “+”, and non-significant changes are denoted by “-”.

According to the above experimental results, a schematic diagram of factors that affected the time interval of repeated collections and the volume at each collection was drawn. As can be seen from the diagram, during repeated CSF collection for a total of four collections, the optimal time interval was 15 days with a volume of 100 μL (Figure 5).

## Discussion

Literature reviews have shown that the total CSF volume from a rat is about 600 μL (22–28). Thus, the volume should not be excessively harvested during collection. An excessive collection volume can easily cause changes in CSF pressure, which could damage sensitive brain tissues. Furthermore, it can also easily lead to CSF blood contamination and death. The results of preliminary experiments showed that a collection volume of less than 150 μL did not cause blood contamination, although it is unknown whether a collection volume of 150 μL has any effect on neurological function. At the same time, given the volume required for CSF sample detection, this experiment examined the safety of collecting a volume of 100 μL and 150 μL. As reviewed in the relevant literature, the average production speed of CSF is 0.4-5.4 μL/min and the turnover rate is 9 days (22–28). Thus, this experiment investigated the safety of the time interval of 10 and 15 d for harvesting CSF specimens.

In the time interval of 10 and 15 d and collecting a volume of 100 and 150 μL, it is unknown whether the collection of CSF leads to marked changes in the cisterna magna pressure. Thus, dynamic changes in cisterna magna pressure after repeated CSF collection are detected. Additionally, CSF samples are mainly used to study the pathogenesis of CNS diseases and drug effects, etc. (29–33).

Therefore, this study investigated the effect of multiple repeated collections of CSF on neurological functions, and dynamic changes in locomotor activity and learning and memory abilities, as well as neurotransmission related to learning and memory as it aligns with the Ach content and CBF in rats (34–40).

The findings of this current study demonstrated that compared with the first CSF collection, pressure decreased in the 10 d-150 μL group after the third collection, which indicated that 10-d repeated collection is not good for rats, even though the CSF sample collected was fine, as Ach levels did not change, locomotor activity was not affected, and cerebral blood flow did not change. A 15 d separation of CSF collection did not show any learning and memory deficit or changes in pressure compared to 10 d, which implied that a longer recovery time between collections is better, especially as CSF turnover rate is 9 d and a lower volume collected is better (100 *vs* 150 μL). In the MWM test, compared with the control groups, the escape latency during the testing period in the 10 d-100 μL and 10 d-150 μL groups was significantly reduced, which indicated that the collection interval of 10 d and a volume of 100 and 150 μL at each collection provoked an effect on both learning and memory. At the same time, in order to further ensure the safety of the CSF collection procedure, the learning process of rats in each group was analyzed, and it showed that the learning process in the 10 d-150 μL group and 15 d-150 μL group was significantly prolonged. There were no significant changes in Ach content, autonomic activity, and cerebral blood flow in each group, showing that CBF collection had no effect on Ach, but it still needs to be tested, which is conducive to the development of a quality control system. Thus, in the process of collecting CSF by multiple repeated percutaneous punctures of the cisterna magna, 100 and 150 μL of CSF collected at intervals of 10 days, and 150 μL of CSF at intervals of 15 days could affect the learning and memory abilities of the rat. Moreover, the risk was increased as the collection volume increased, which might be a major consideration during collection. Therefore, the recommended time interval and collection volume for CSF harvesting was 15 d and 100 μL, respectively. In future studies, the pathological changes of the brain tissue should be measured.

In conclusion, this study systematically investigated the safety of multiple repeated percutaneous punctures of the cisterna magna with the intention of collecting CSF. We determined the optimal time interval and volume for collection, which thus provided a method for multiple repeated CSF harvests that were characterized by a high degree of safety and strong or robust ease of operability.
